# Registros e Medicina Baseada em Evidências

**DOI:** 10.36660/abc.20240330

**Published:** 2024-07-15

**Authors:** Fernando Antibas Atik

**Affiliations:** 1 Instituto de Cardiologia e Transplantes do Distrito Federal Brasília DF Brasil Instituto de Cardiologia e Transplantes do Distrito Federal, Brasília, DF – Brasil

**Keywords:** Cirurgia Cardíaca, Revascularização do Miocárdio, Complicações

Desde a conquista histórica de René Favaloro em 1967, a cirurgia de revascularização do miocárdio (CRM) desenvolveu grandes avanços.^1^ Apesar da ampla adoção da intervenção coronária percutânea com implante de stent, a CRM continua sendo uma opção de tratamento invasivo extremamente importante em pacientes com doença arterial coronariana. A cirurgia de revascularização miocárdica tem sido extensivamente investigada na literatura por meio de muitos ensaios clínicos randomizados e registros observacionais multicêntricos e unicêntricos em grande escala, que proporcionaram adequação de decisão em uma abordagem centrada no paciente, alcançando o mais alto nível de recomendação em muitos cenários clínicos.

Neste número dos Arquivos Brasileiros de Cardiologia, o manuscrito intitulado "Idade, Insuficiência Renal e Transfusão são Preditores de Risco de Longa Permanência Hospitalar após Cirurgia de Revascularização do Miocárdio"^2^ aborda uma questão importante. Os autores tiveram como objetivo determinar os fatores associados ao tempo prolongado de internação pós-operatória, definido como superior a 14 dias. Os autores estudaram 3.703 pacientes submetidos à CRM durante 2 anos, utilizando o Registro Estadual de São Paulo REPPLICAR II. Esses dados estão desatualizados em uns 5 anos. Encontraram que 6,16% dos pacientes tiveram tempo de internação pós-operatória prolongado, sendo os determinantes pré e intraoperatórios do desfecho primário, por meio de análise de regressão logística multivariada, presença de idade superior a 60 anos, disfunção renal pré-operatória e transfusão intraoperatória de hemácias.

Por que é tão importante estudar isso? A predição de risco é importante na prática médica, pois permite comparações objetivas entre instituições e cirurgiões para ajustar as características da gravidade da doença. Além disso, os escores de risco pré-operatório são úteis no esclarecimento do consentimento pré-operatório, no controle de qualidade dos serviços e na seleção ou exclusão de pacientes em ensaios controlados. Mais especificamente, o tempo prolongado de internação pós-operatória está relacionado a mais eventos de morbidade e está associado a maiores custos. O conhecimento dos fatores associados ao maior tempo de internação pode ser utilizado no direcionamento de protocolos hospitalares para mitigar esse problema. Além disso, seria a base de reembolso para sistemas de desempenho em todos os sistemas de prática pública e privada, em vez do atual método de pagamento de taxas que é a realidade na maioria dos hospitais brasileiros.

Os dados do manuscrito são provenientes de do Registro Estadual de São Paulo REPPLICAR II. Um registro de pacientes é um sistema organizado que utiliza métodos de estudo observacional para coletar dados uniformes (clínicos e outros) para avaliar resultados específicos para uma população definida por uma doença, condição ou exposição específica, e que atende a um ou mais propósitos científicos, clínicos ou de política. Ensaios clínicos randomizados fornecem um nível de evidência mais elevado do que estudos observacionais. No entanto, os critérios de exclusão desses estudos limitam a sua generalização, sendo estudos observacionais de registros nacionais mais voltados para a prática médica do mundo real. Embora os registros possam fornecer informações úteis, existem níveis de rigor que aumentam a validade e tornam as informações de alguns registros mais úteis para orientar decisões. Os registros de pacientes que observam a prática clínica do mundo real podem recolher as informações necessárias para avaliar os resultados dos pacientes de uma forma generalizável, mas a interpretação correta destas informações requer uma metodologia analítica orientada para abordar as potenciais fontes de viés que desafiam os estudos observacionais. A interpretação dos dados do registro de pacientes também requer verificações da validade interna e, por vezes, a utilização de fontes de dados externas para validar os principais pressupostos.

Os autores devem ser elogiados pelo esforço e sucesso no desenvolvimento e manutenção de um registro multicêntrico de cirurgia cardíaca em nosso país, com suas limitações herdadas para fazê-lo.^3^ Isto não é novidade. Entre 2015 e 2018, a Sociedade Brasileira de Cirurgia Cardiovascular criou o Registro BYPASS que publicou os primeiros resultados de CRM em escala nacional.^4^ Infelizmente, o Registro BYPASS foi descontinuado, mas ajudou a criar uma rede de comunicação entre os diferentes centros do país. Felizmente, um Banco de Dados Nacional de CRM chamado BRASCORE foi lançado oficialmente em 2024 e certamente será uma importante fonte de informações em breve. Os países desenvolvidos têm tido mais sucesso do que nós neste aspecto, particularmente na organização social, no financiamento específico da base de dados, no compromisso individual dos centros com a base de dados e, em alguns casos, na submissão obrigatória da base de dados associada ao pagamento. Por exemplo, o banco de dados de cirurgia cardíaca da Sociedade de Cirurgiões Torácicos tem sido uma fonte contínua de informações há mais de 20 anos, gerando evidências relevantes sobre questões importantes, com inúmeras publicações de alto impacto na literature.^5^

Embora os dados do artigo^2^ sejam oriundos de um importante Registro Estadual, existem algumas limitações comuns dos registros que devem ser mencionadas. Muitas vezes incluem qualidade variável dos dados, falta de detalhes nos dados coletados que geralmente não são feitos pelo pesquisador e informações confusas. Além disso, a falta de informação é comum, bem como diferentes abordagens são utilizadas para lidar com dados faltantes que não são relatados. No manuscrito publicado nos Arquivos Brasileiros de Cardiologia,^2^ aproximadamente 8,5% dos sujeitos foram excluídos por falta de informações sobre o tempo de internação hospitalar. Além disso, a proposta de pesquisa com seu desenho surge após a criação dos dados cadastrais. Ao fazer isso, variáveis importantes relevantes para a questão de pesquisa ficam indisponíveis. Outros fatores que devem ser levados em consideração são o número de centros e a sua contribuição relativa para o Registro, a heterogeneidade do volume do centro, a expertise e os protocolos de manejo intraoperatório e cuidados intensivos pós-operatórios. Por essas razões, os resultados do manuscrito devem ser interpretados com cautela e, duvidosamente, traduzidos para nível nacional.

A discussão sobre a hospitalização prolongada após um procedimento cirúrgico de grande porte é muito importante e os autores estão de parabéns por fazê-la. Para chegar a conclusões significativas, seria importante determinar as razões associadas à hospitalização prolongada, juntamente com os seus preditores. Problemas clínicos comuns incluem fibrilação atrial de início recente, complicações pulmonares, complicações neurológicas, lesão renal aguda e infecção de feridas, entre outros.
