# Supplementary material for: Registros e Medicina Baseada em Evidências
Source: Arq Bras Cardiol. 2024 Jul 15;121(6):e20240330. [Article in Portuguese] doi: 10.36660/abc.20240330 (PMC12092039; doi:10.36660/abc.20240330)
Supplement: Translation

# Translation: Registries and Evidence-Based Medicine

**DOI:** 10.36660/abc.20240330i

**Authors:** Fernando Antibas Atik

**Affiliations:** 2 Brasília DF Brazil Instituto de Cardiologia e Transplantes do Distrito Federal, Brasília, DF – Brazil

**Keywords:** Heart Surgery, Coronary Artery Bypass Grafting, Complications

Since the landmark achievement of Rene Favaloro in 1967, coronary artery bypass grafting (CABG) has developed great advances.^1^Despite the widespread adoption of percutaneous coronary intervention with stenting, CABG remains an extremely important invasive treatment option in patients with coronary artery disease. Coronary artery bypass grafting has been extensively investigated in the literature through many randomized controlled trials and large-scale multicenter and unicenter observational registries, which have provided appropriateness of decision in a patient-centered approach, achieving the highest level of recommendation in many clinical scenarios.

In this issue of *Arquivos Brasileiros de Cardiologia,* a manuscript entitled "Age, Renal Failure and Transfusion are Risk Predictors of Prolonged Hospital Stay after Coronary Artery Bypass Grafting Surgery"^2^addresses an important issue. The authors aimed to determine factors associated with prolonged postoperative hospital length of stay, defined as greater than 14 days. The authors studied 3703 patients submitted to CABG over 2 years, using the Sao Paulo State Registry REPPLICAR II. This data is out of date in about 5 years. They have found that 6.16% of patients had prolonged postoperative length of stay, being the preoperative and intraoperative determinants of the primary outcome, using multivariable logistic regression analysis, the presence of age greater than 60 years, preoperative kidney dysfunction, and intraoperative red blood cell transfusion.

Why is so important to study this? Risk prediction is important in medical practice, as it allows objective comparisons between institutions and surgeons to adjust the characteristics of the severity of disease. Moreover, the scores of preoperative risk are useful in clarifying the preoperative consent, in quality control of services, and in the selection or exclusion of patients in controlled trials. More specifically, prolonged postoperative length of stay relates to more morbidity events, and it is associated with greater cost. The knowledge of factors associated with greater hospital stays may be used in targeting hospital protocols to mitigate this problem. Additionally, it would be the basis of reimbursement for performance systems throughout the public and private practice systems, instead of the current method of fee payment that is the reality in most Brazilian hospitals.

The manuscript data comes from the Sao Paulo State Registry REPPLICAR II. A patient registry is an organized system that uses observational study methods to collect uniform data (clinical and other) to evaluate specified outcomes for a population defined by a particular disease, condition, or exposure, and that serves one or more scientific, clinical, or policy purposes. Randomized controlled trials provide a higher level of evidence than observational studies. However, the exclusion criteria of those studies limit their generalizability, being observational studies from national registries more attained to real-world medical practice. Although registries can provide useful information, there are levels of rigor that enhance validity and make the information from some registries more useful for guiding decisions. Patient registries that observe real-world clinical practice may collect the information needed to assess patient outcomes in a generalizable way, but interpreting this information correctly requires analytic methodology geared to address the potential sources of bias that challenge observational studies. Interpreting patient registry data also requires checks of internal validity and sometimes the use of external data sources to validate key assumptions.

The authors should be commended for their effort and success in developing and maintaining a multicenter cardiac surgery registry in our country, with its inherited limitations to do so.^3^This is not novel. Between 2015 and 2018, The Brazilian Society of Cardiovascular Surgery created the BYPASS Registry which has published the first results of CABG on a national scale.^4^Unfortunately, the BYPASS Registry was discontinued, but it helped to create a communication network among different centers across the country. Fortunately, a Brazilian National CABG Database called BRASCORE was officially launched in 2024, and it will certainly be an important source of information soon. Developed countries have been more successful than us on that, particularly in societal organization, specific database funding, individual center commitment to the database, and in some instances mandatory database submission that is linked to payment. For instance, the Society of Thoracic Surgeons cardiac surgery database has been an ongoing source of information for more than 20 years, generating relevant evidence about important issues, with numerous high-impact publications in the literature.^5^

Although the manuscript^2^data comes from an important State Registry, there are some common limitations of registries that should be mentioned. They often include variable quality of data, lack of detail in the data collected that is usually not done by the researcher, and confounder information is lacking. Moreover, missing information is common, as well as different approaches are used to deal with missing data that is not reported. In the manuscript published at *Arquivos Brasileiros de Cardiologia* ,^2^approximately 8.5% of the subjects were excluded due to a lack of information about hospital length of stay. Additionally, the research proposal with its design comes after the creation of the registry data. By doing so, important variables that are relevant to the research question are unavailable. Other factors that should be taken into account are the number of centers and their relative contribution to the Registry, heterogeneity of center volume, expertise, and protocols of intraoperative management and postoperative intensive care. For those reasons, the results of the manuscript should be interpreted with caution, and doubtfully being translated to a national level.

Discussion about prolonged hospitalization after a major surgical procedure is very important and the authors are congratulated for doing it. To achieve meaningful conclusions, it would be important to determine the reasons associated with prolonged hospitalization, along with their predictors. Common clinical problems include new-onset atrial fibrillation, pulmonary complications, neurologic complications, acute kidney injury, and wound infection, among others.
